# Analysis of in vitro profiling data of cosmetic ingredients within the Tox21 10K compound library for bioactivity and potential toxicity

**DOI:** 10.1186/s40360-026-01165-5

**Published:** 2026-06-16

**Authors:** Laken Kruger, Deborah K. Ngan, Tuan Xu, Menghang Xia, Stephen S. Ferguson, David M. Reif, Anton Simeonov, Ruili Huang

**Affiliations:** 1 Division of Preclinical Innovation (DPI), National Center for Advancing Translational Sciences (NCATS), National Institutes of Health (NIH), 9800 Medical Center Drive, Rockville, MD 20850 USA; 2 Division of Translational Toxicology, National Institute of Environmental Health Sciences (NIEHS), National Institutes of Health (NIH), Research Triangle Park, NC USA

**Keywords:** Cosmetics, Tox21, In vitro assay, Phthalates, PFAS

## Abstract

**Background:**

Cosmetics are defined by the U.S. Food and Drug Administration (FDA) as “articles intended to be rubbed, poured, sprinkled, or sprayed on, introduced into, or otherwise applied to the human body…for cleansing, beautifying, promoting attractiveness, or altering the appearance”. However, the safety of cosmetic ingredients is the responsibility of the manufacturer and exposure to some of these chemicals can result in unintentional harmful effects in humans. Thus, some states are banning specific chemicals, at the state legislation level, from being used in cosmetics, which includes known endocrine disruptors such as dibutyl phthalate, diethylhexyl phthalate, and per- and polyfluoroalkyl substances (PFAS). In this study, we aim to determine the toxicity pathways significantly affected by the cosmetic ingredients (both banned and non-banned), compared to the non-cosmetics compounds in the Tox21 10K library.

**Methods:**

The Tox21 10K compound library (which includes 113 banned, 927 non-banned cosmetic ingredients and 8,185 non-cosmetic compounds) was previously screened against a panel of ~ 90 cell-based and biochemical assays. The hit rates (i.e., the percentage of compounds active in an assay) of the cosmetic and non-cosmetic compounds, as well as banned and non-banned compounds, in each Tox21 assay were compared using a Fisher’s exact test, and a p-value < 0.05 was considered statistically significant.

**Results:**

We found that the evaluated cosmetic compounds were significantly more active in 11 antagonist mode assays and 23 agonist mode assays compared to the non-cosmetic compounds. The banned compounds were significantly more active in 7 antagonist mode and 6 agonist mode assays compared to the non-banned compounds. The targets of the antagonist assays included enzymes such as CYP2C19 and aromatase (CYP19A1), and the agonist assays included the Keap1/Nrf2 antioxidant response element pathway.

**Conclusions:**

In the present study, we compared the bioactivity profiles of cosmetic ingredients and non-cosmetic compounds (not used in cosmetics), as well as the banned and non-banned cosmetic ingredients, across the Tox21 assays. The analyses revealed distinct molecular pathways for these chemicals which furthers our mechanistic understanding of cosmetics ingredient toxicity and may help direct the selection of cosmetic ingredients in the future with potentially less bioactivity.

**Clinical trial number:**

Not applicable.

**Supplementary Information:**

The online version contains supplementary material available at 10.1186/s40360-026-01165-5.

## Background

The U.S. Food and Drug Administration (FDA) defines cosmetics as “articles intended to be rubbed, poured, sprinkled, or sprayed on, introduced into, or otherwise applied to the human body...for cleansing, beautifying, promoting attractiveness, or altering the appearance” in the Federal Food, Drug & Cosmetic Act (FD&C Act) Section  201(i) [1]. Since cosmetics are a common part of everyday life, monitoring and regulating the ingredients is crucial to protecting the population from unnecessary harm. Unlike the European Union which uses a proactive approach based on pre-market notification, safety assessment, and post-market analysis in cosmetics production, the United States only uses post-market analysis. Under the U.S. laws, as of 2022, FDA approval is not required for cosmetic ingredients or products before they become available on the market and it is considered the company’s legal obligation to ensure product safety [1]. Currently, the FDA only prohibits or restricts the use of 11 compounds, or classes of compounds, such as mercury-containing compounds compared to over 2500 being banned in the EU [2, 3]. Since there is limited oversight at the federal level, 19 states have banned (e.g., prohibited the use) or begun banning chemicals in cosmetic products sold or produced in their state, such as California. First introduced in 2023, California proposed a bill to ban certain compounds from being utilized as cosmetic ingredients [4]. The bill was enacted on January 1, 2025 and has banned 26 chemical classes (70 total compounds in the Tox21 compound library) from being used in cosmetics products that are sold or produced in the state, which include a couple phthalates and per- and polyfluoroalkyl substances (PFAS) [4]. Eighteen other states have also submitted bills banning certain compounds or classes of compounds from being used as cosmetic ingredients (113 total banned compounds in the Tox21 compound library), which also includes PFAS (16 states) and phthalates (Georgia, Maryland, and New York) [5].

Phthalates comprise a broad class of compounds, some of which are known endocrine disruptors that can interfere with hormone signaling pathways. They are commonly utilized as plasticizers to make fragrances last longer or make plastics more flexible. Two of the most widely used phthalates, dibutyl phthalate (DBP) and diethylhexyl phthalate (DEHP), were banned at the state level (California, Georgia, Maine, Maryland, and New York). DBP and its metabolites have recently been linked to impaired sperm function in mice [6] and in utero exposure in rats has been linked to hypospadias [7] and neuroendocrine system disruption over multiple generations [8]. DEHP and its metabolites have been shown to affect immune function [9] as well as alter the placenta proteome and transcriptome [10, 11]. In utero exposure to phthalates has also been associated with altered fetal development like decreased anogenital distance [12] and childhood conditions such as asthma [13].

Another banned class of compounds is per- and polyfluoroalkyl substances (PFAS), which are a class of persistent synthetic chemicals found in the blood of 97% of Americans [14] Unlike phthalates, the PFAS were banned as a whole class while phthalates started being banned individually. PFAS are used in a variety of fields ranging from aerospace to textiles [15] and are often referred to as “forever chemicals” due to their stable C-F bonds that allow them to persist in the environment without degradation for an extended period of time [16]. While some PFAS affect multiple pathways and targets, including the Sonic Hedgehog (SHH) pathway, which plays a critical role in neurogenesis and embryonic development, as well as the synthesis of cholesterol, steroids, and other lipids (via CYPs such as CYP2C9) [17], other PFAS have minimal to no effect [16]. PFAS have recently been linked to neurotoxicity via accumulation in the brain leading to neurotransmitter alterations, structural neuron changes, and cognitive disorders [18–20]. Recent studies have also linked PFAS exposure to increased risk of cancers such as kidney, ovary, prostate, testicle, and thyroid, [14, 21–23] as well as altering reproductive and development systems [24, 25]. PFAS have also been shown to alter the metabolomic profile of human milk [26].

Though phthalates and PFAS are known to disrupt specific pathways, like many banned compounds, there are many non-banned compounds that are used in cosmetics with unknown potential for human toxicity. The aim of the Toxicology in the 21st Century (Tox21) program [27–32] is to improve the efficiency of toxicity prediction and testing. Tox21 has developed in vitro methods to efficiently evaluate the potential toxicity of compounds and has previously tested about 10,000 chemical samples in the “Tox21 10K library” utilizing ~ 90 cell-based and biochemical assays in a quantitative high throughput screening (qHTS) format [28, 32]. This testing has resulted in over 122 million data points that are publicly available [33, 34]. The Tox21 assays primarily utilized human cell lines (74.3%) and covered multiple toxicity-related pathways including nuclear receptor signaling, stress response pathways, G-protein coupled receptor signaling, neurotoxicity, genotoxicity, developmental toxicity and metabolism [32]. There are a variety of uses for the chemicals in the Tox21 10K library, including cosmetic ingredients. Understanding the potential mechanisms and pathways that are associated with the toxicity of cosmetic ingredients may help select ingredients with limited bioactivity and a more favorable safety profile. In the present study, we analyzed the compounds that were banned as cosmetic ingredients across multiple states, [4, 5] as well as compounds classified as cosmetic ingredients by the U.S. Environmental Protection Agency (EPA) within the Tox21 10K compound library to determine the pathways affected by cosmetic ingredients.

## Methods

### Compound library

**Fig. 1:**
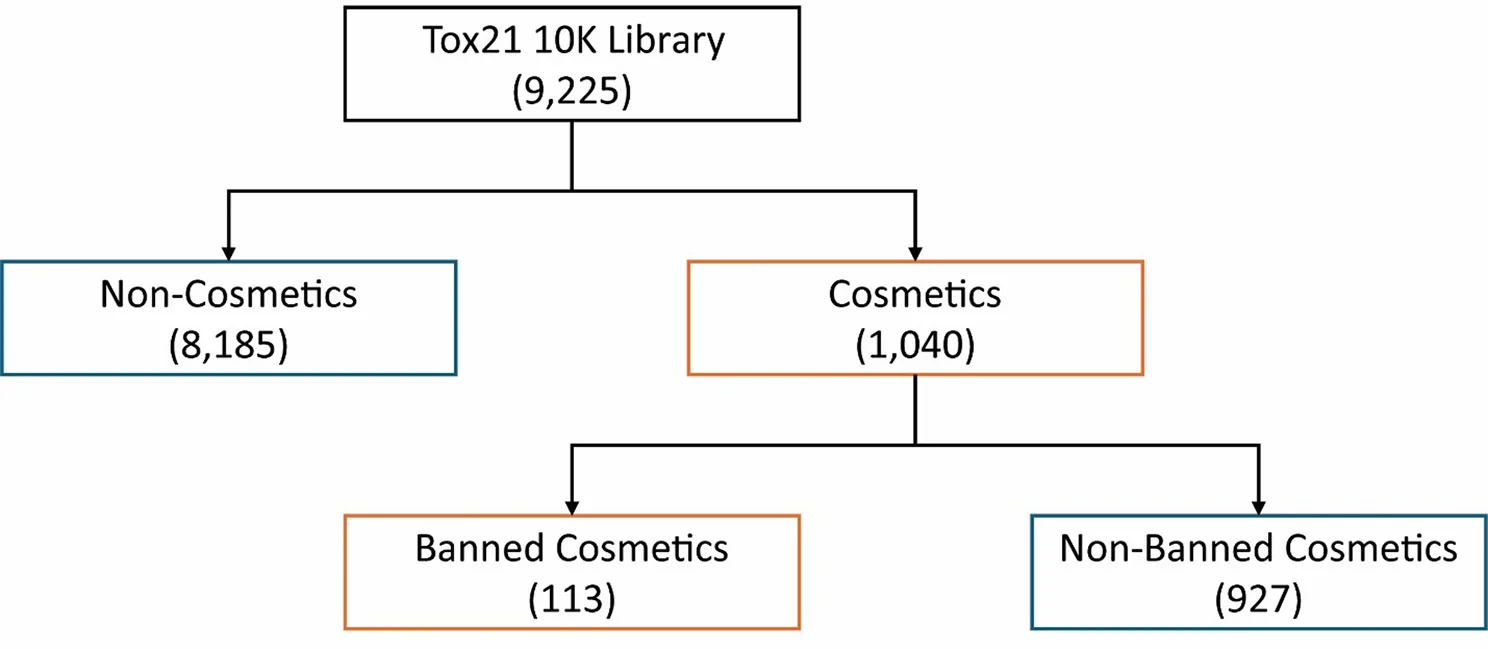
Flowchart of how the compounds were binned for analysis from the Tox21 10K library

The Tox21 10K chemical library consists of about 10,000 (9,225 unique) compounds provided by the U.S. EPA in addition to compounds from the National Toxicology Program (NTP) of the National Institute of Environmental Health Sciences (NIEHS) and the National Center for Advancing Translational Sciences (NCATS) Pharmaceutical Collection (NPC) [28, 35, 36]. The Tox21 10K library includes drugs and environmental chemicals such as pesticides, food additives, consumer products, industrial chemicals, and cosmetic ingredients [28]. This library consists of 972 compounds that were classified by the U.S. Environmental Protection Agency (EPA) Chemical and Product Categories Database (CPCat) as cosmetic ingredients or used in cosmetic products [37, 38]. The EPA uses data from a variety of available resources including government and industry sources to determine compound category [39]. This database does not update the labels when a compound is removed from a category so the categorization is historical. Importantly, the classification of chemicals in Tox21 is not singular and a chemical that is classified as “cosmetic” may also be classified a “drug” and vice versa. A total of 1,040 cosmetic compounds were included for analysis in this study due to some banned cosmetic ingredients [5] not being labeled as such on the EPA CPCat list (Supplementary Table 1). Banned cosmetic ingredients (113 in the Tox21 10K library) were classified as chemical compounds that were either banned or in the process of being banned at the state level at the time of this study; this includes compounds that fall into the classes of banned chemicals such as mercury containing compounds. Non-banned cosmetic ingredients (927 in the Tox21 10K library) are compounds that were identified as cosmetic ingredients but are not on any banned list. The remaining compounds (8,185) in the Tox21 library that were not labeled as cosmetic ingredients were considered non-cosmetics. A flowchart of the compound binning is shown in Fig. 1. Further analysis included 1425 pesticides [40] and 3765 drug [41] compounds within the Tox21 library.

### In vitro assay data

The Tox21 10K compound library has been screened against ~ 90 assays covering a wide range of targets and pathways such as nuclear receptor signaling (NR, 48.7%), stress response (SR, 10.3%), metabolism (6.4%), G-protein coupled receptors (GPCR, 5.1%), cytotoxicity (7.7%), and other toxicologically relevant targets/pathways (17.9%). The cell lines that were utilized in these qHTS assays included human cell lines (74.3%), murine embryo fibroblast (6.4%), Chinese hamster ovary cell lines (5.1%), biochemical (10.3%), and other (3.9%). Tox21 assay data is publicly available on https://tripod.nih.gov/pubdata/ [33] and PubChem [34].

The Tox21 compound activity in each assay was measured by first assigning a class based on the concentration-response curve observed (1.1, 1.2, 1.3, 1.4, 2.1, 2.2, 2.3, 2.4, and 3 for actives; 4 for inactive). The curve class was then converted into a curve rank, an integer between − 9 and 9, where a higher absolute rank corresponds to a higher curve quality, potency, and efficacy according to previously described criteria [42, 43]. The compound was then assigned an activity outcome as previously described, based on the average curve rank and reproducibility from the triplicate assay runs, as one of the following: active agonist/antagonist, inconclusive agonist/antagonist, inconclusive, or inactive [42, 43]. Compound potency was measured by the half maximal activity (AC_50_) based on the concentration-response curve. Many of the Tox21 assays were coupled with a cell viability counter screen (CellTiter-Glo, CellTiter-Fluor, and real-time cytotoxicity) as an indicator of cytotoxicity.

### Data analysis

The compound hit rates (percentage of active compounds in an assay) between the banned and non-banned cosmetics ingredients, then cosmetics vs. non-cosmetic compounds, then cosmetics vs. pesticides, then cosmetics vs. drugs were compared in each assay using a Fisher’s exact test performed within R version 4.1.2. The Fisher’s exact test is a statistical test that examines the relationship between two groups to determine the statistical significance. A p-value < 0.05 was considered statistically significant. The assay hit rates (i.e., the percentage of Tox21 assays hit by a compound) of the cosmetic and non-cosmetic compounds, as well as banned and non-banned compounds, were compared further using a box and whisker plot. The compounds in the Tox21 10K library were clustered based on chemical structure (represented as Extended Connectivity Fingerprints (ECFP4)) similarity using the self-organizing map (SOM) algorithm [44, 45], resulting in 653 clusters. The SOM was used for easy visualization, and the clusters are labeled as “column.row”, e.g., k1.1 represents column 1 row 1. Each cluster was evaluated for the enrichment of banned and non-banned cosmetic compounds compared to library average. Statistical significance was determined by the Fisher’s exact test.

## Results

### Bioactivity profiles of cosmetic ingredients across the Tox21 assays

Compounds in the Tox21 10K library reported as cosmetic ingredients (1,040), along with non-cosmetic compounds (8,185) were screened against ~ 90 in vitro assays in a high throughput concentration-response format. The assays were run in two modes: agonist mode that was designed to detect compounds that activate or induce a biological target or pathway and antagonist mode that was designed to detect inhibitors of a target or pathway. The frequencies of active compounds (antagonists or agonists) among different groups, e.g., banned and non-banned compounds, were compared in each assay to determine which targets or pathways were significantly affected by the banned compounds within the Tox21 10K library.

**Fig. 2:**
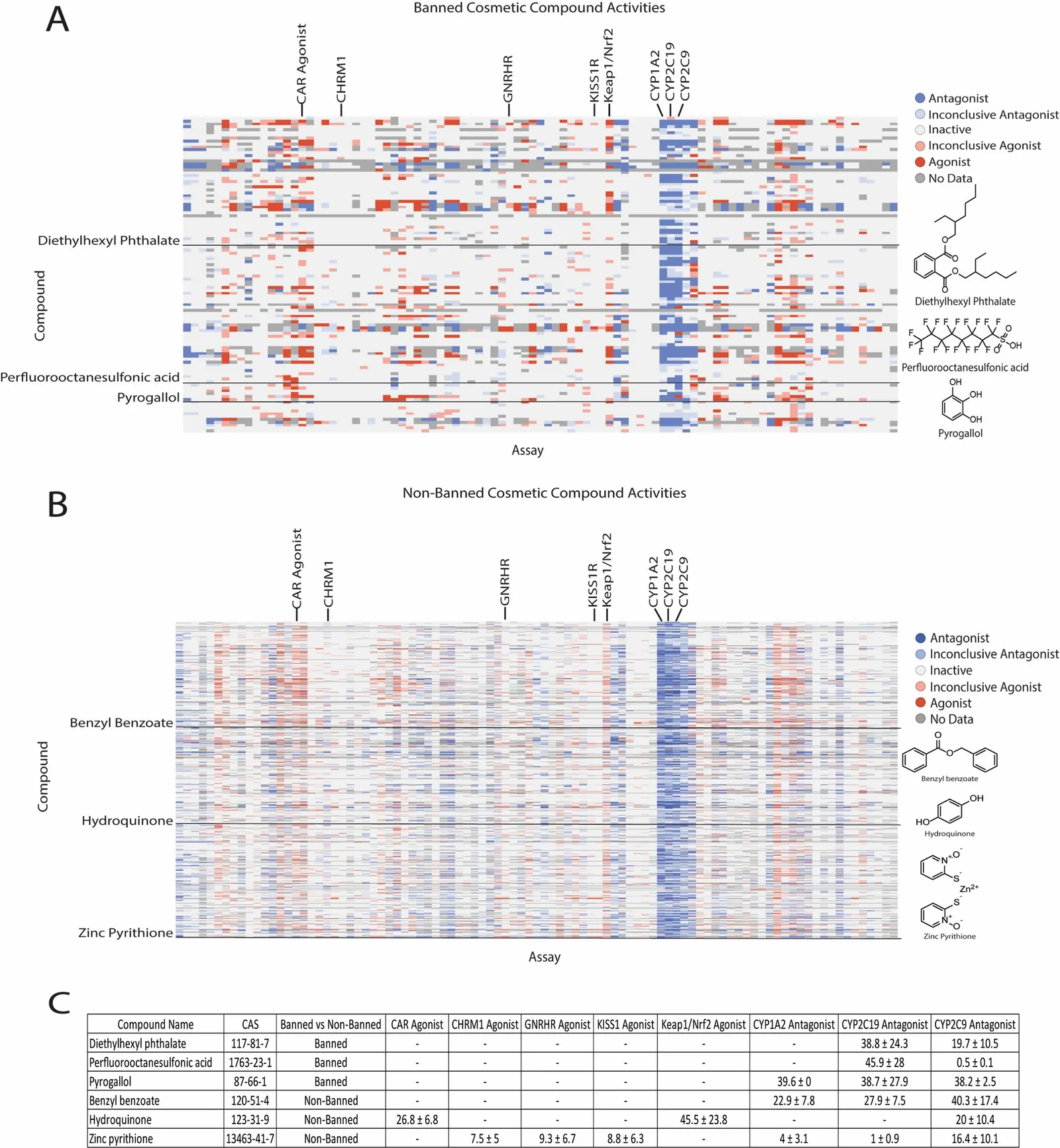
Heatmaps of Banned (**A**) and Non-banned (**B**) compound activities across the Tox21 assay panel. In the heatmaps, each row is a compound, and each column is an assay. The heatmaps are colored by compound activity, which can be categorized into: “active agonist”, “inconclusive agonist”, “active antagonist”, “inconclusive antagonist”, “inconclusive”, and “inactive”. A dark red color indicates a strong activator, a dark blue color indicates a strong inhibitor, inactive compounds are colored in white, and dark gray indicates inconclusive data. Significant assays are labeled, and example compounds are highlighted on the right. The compounds (columns) and assays (rows) are arranged in alphabetical order in the heatmap. The heatmaps are accompanied by a table (**C**) of the average AC_50_ and standard deviation, in µM, of the example compounds in the highlighted assays

Out of the 1040 cosmetic ingredients, 113 banned compounds and 927 non-banned compounds were detected (Supplementary Table 1). The bioassay activities of the banned and non-banned cosmetic compounds screened against the Tox21 assay panel were summarized in heatmaps (Fig. 2). Each row of the heatmap represents a banned cosmetic ingredient (Fig. 2A) or a non-banned cosmetic ingredient (Fig. 2B) and each column represents one of the Tox21 activity assays. The heatmaps are color coded where the blue represents active antagonists, red represents active agonists, white represents inactive, and gray represents no data. Both the banned and non-banned compounds showed a broad range of activities across the Tox21 assay panel, indicating that the compounds interfered with multiple pathways as potential hazards for human health. The assays highlighted in Fig. 2 are ones where cosmetic ingredients (banned or non-banned) were significantly more active compared to the non-cosmetic compounds. For example, a large fraction of cosmetic ingredients was found to be active antagonists (blue) of CYP1A2, CYP2C9, or CYP2C19, or active agonists (red) of CHRM1, GnRHR, or Keap1/NrF2. The average AC_50_ and standard deviation for the example compounds in the highlighted assays were compiled into a table (Fig. 2C) to show the potency of the compounds relative to each other. Interestingly, a non-banned compound, Zinc Pyrithione, was the most promiscuous compound with the lowest average AC_50_ across the highlighted assays. Zinc Pyrithione is already banned in the European Union as of 2022 due to its classification as a reproductive toxicant^2^ but is not banned in the United States and can be found in antidandruff shampoos.

**Fig. 3:**
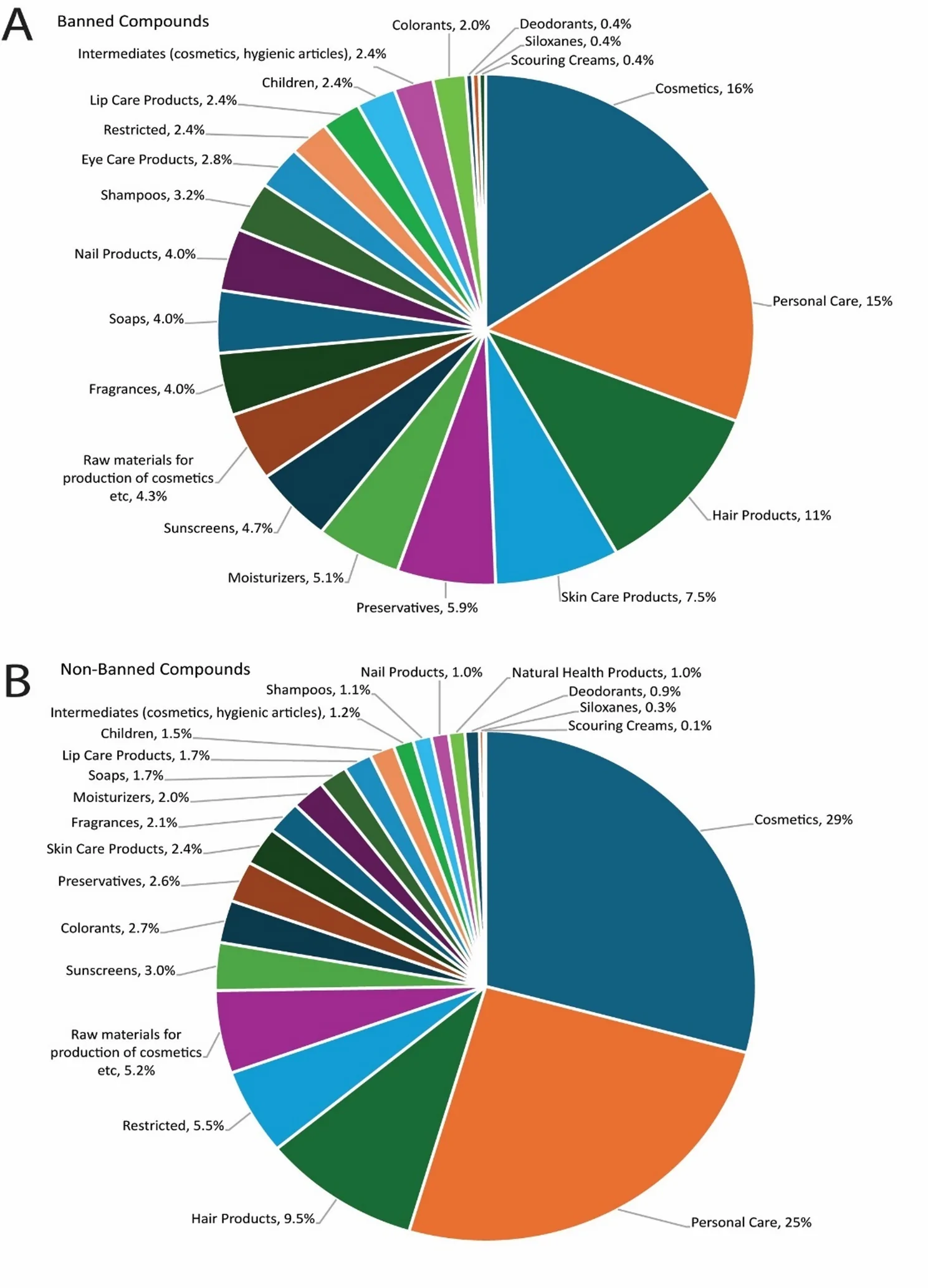
Product use category distributions of the banned (**A**) and non-banned (**B**) cosmetic compounds

These cosmetic ingredients (both banned and non-banned compounds) are found in a variety of products. The distribution of product types that the cosmetic ingredients are used in are visualized in Fig. 3. There were 22 categories of product uses, including generalized terms “Cosmetics” and “Personal Care”, listed in the EPA CPCat (Chemical and Product Categories Database) which show current and historical uses of the product. Since the EPA CPCat does not remove categorizations, a few chemicals have historic classifications. The pie charts show that both the banned compounds and non-banned compounds were mainly present in hair care products (11% and 9.5%, respectively). While the second highest category for the banned and non-banned compounds were skin care products (7.5%) and compounds restricted for public use (5.5%), respectively. The third highest categories were “preservatives” (5.9%) for the banned compounds and “raw materials for the production of cosmetics” (5.2%) for the non-banned compounds. This vast distribution in product type categories shows that the exposure that we get from these compounds vary greatly in the source and amount.

**Fig. 4:**
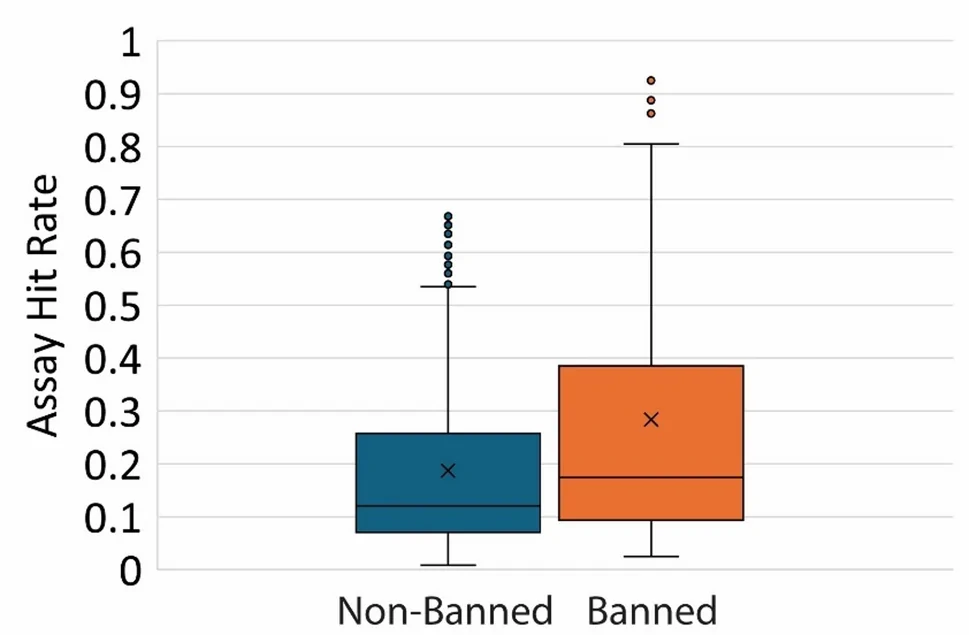
Distribution of the assay hit rates of the banned and non-banned cosmetic compounds

When the assay hit rate (percentage of assays the compound is active in) of the non-banned and banned cosmetic ingredients were compared further, the banned compounds showed a slightly wider range of activities (Fig. 4). The median assay hit rates were 17.43% and 12.04% for the banned and non-banned compounds, respectively. The banned compounds had a higher interquartile range of 28.95% compared to the non-banned compounds at 24.48%. When the cell viability assay hit rates (a indicator of cytotoxicity) were compared between the banned and non-banned compounds, the banned compounds showed a wider range with an interquartile range of 35.29% compared to the non-banned at 27.06%. The median cell viability assay hit rates were 12.94% and 9.41% for the banned and non-banned compounds, respectively. The top 20 most promiscuous and cytotoxic cosmetic ingredients are listed in Table 1.

**Table 1:** Top 20 most promiscuous and cytotoxic cosmetic compounds

Compound Name	CAS	Viability Assay Hit Rate	Tox21 Assay Hit Rate	Banned vs. Non-Banned
Phenylmercuric acetate	62-38-4	96.5%	89.2%	Banned
Mercury(II) acetate	1600-27-7	97.6%	88.8%	Banned
Phenylmercuric chloride	100-56-1	94.1%	87.6%	Banned
Thimerosal	54-64-8	95.3%	87.1%	Banned
Mercury(II) iodide	7774-29-0	97.6%	86.3%	Banned
N-Methyl-p-aminophenol sulfate	55-55-0	95.3%	86.3%	Non-Banned
N, N-Dimethyl-N-benzyl-N-octadecylammonium chloride	122-19-0	96.5%	85.9%	Non-Banned
Benzyl-C12-18-alkyldimethylammonium chlorides	68391-01-5	96.5%	85.9%	Non-Banned
Benzalkonium chloride	8001-54-5	96.5%	84.6%	Non-Banned
Chlorhexidine dihydrochloride	3697-42-5	97.6%	84.2%	Non-Banned
Benzyl-C8-18-alkyldimethylammonium chlorides	63449-41-2	95.3%	84.2%	Non-Banned
Myristyltrimethylammonium chlorideN, N,N-Trimethyltetradecan-1-aminium chloride	4574-04-3	89.4%	83.4%	Non-Banned
Zinc pyrithione	13463-41-7	90.6%	83.0%	Non-Banned
Thiram	137-26-8	92.9%	82.6%	Non-Banned
N, N,N-Trimethyloctadecan-1-aminium chloride	112-03-8	94.1%	82.2%	Non-Banned
Chlorhexidine	55-56-1	94.1%	81.7%	Non-Banned
Chlorhexidine diacetate	56-95-1	94.1%	81.7%	Non-Banned
Benzethonium chloride	121-54-0	96.5%	81.3%	Non-Banned
Hexadecyltrimethylammonium bromide	57-09-0	91.8%	80.5%	Non-Banned
Acetaldehyde	75-07-0	91.8%	80.5%	Banned

### Tox21 assays significantly enriched with active cosmetic compounds

**Fig. 5:**
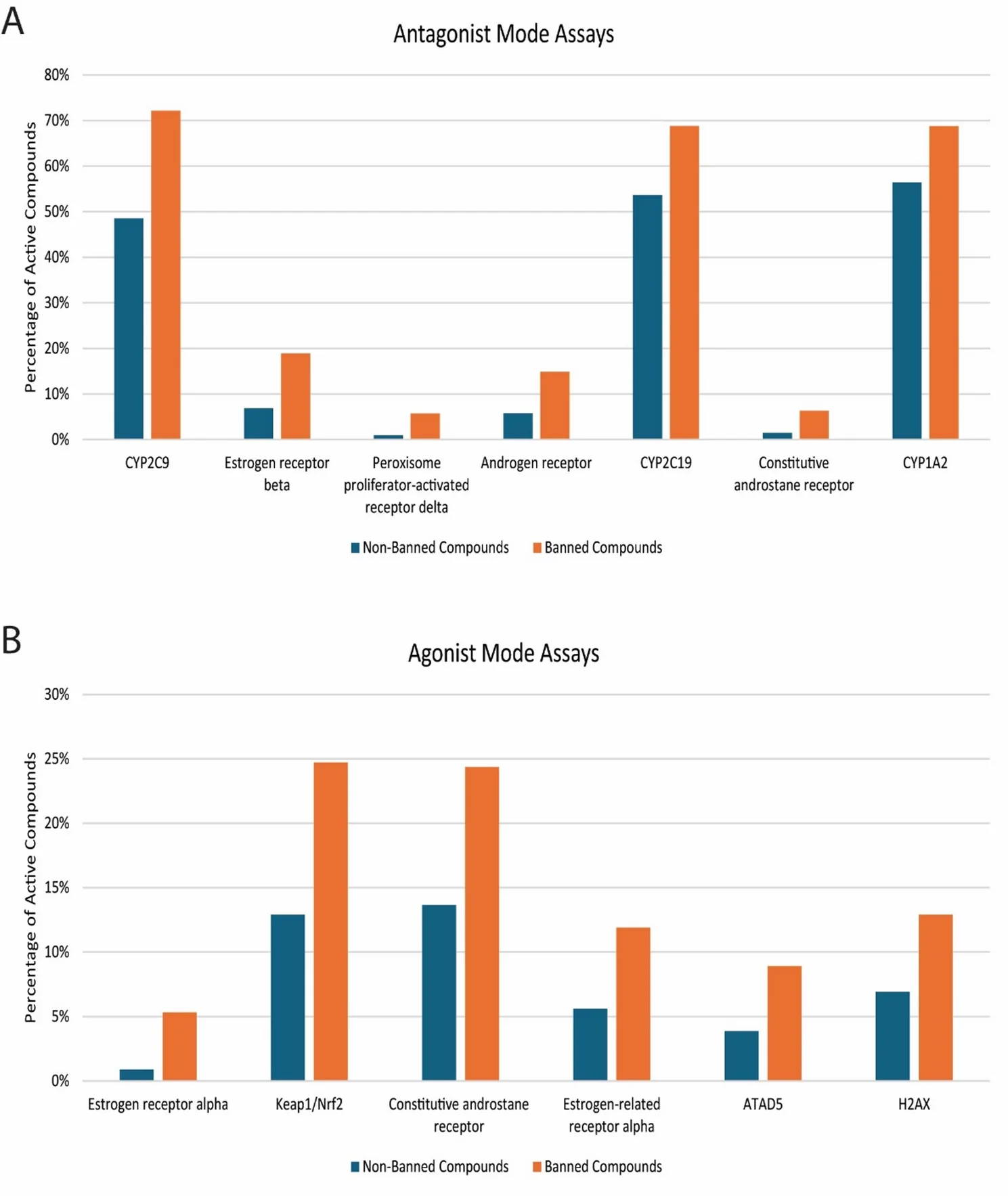
The percentage of active compounds in the banned and non-banned groups in antagonist mode (**A**) and agonist mode (**B**) assays significantly altered by banned cosmetic compounds

To identify the specific biological targets or pathways that the banned compounds may disrupt, the active compound hit rates (percentage of active compounds in an assay) between the banned and non-banned cosmetics ingredients were compared in each assay. Seven antagonist mode and six agonist mode assays were found to be significantly enriched with actives in the banned cosmetic ingredients compared to the non-banned compounds (Table 2). For example, the banned cosmetic ingredients had significantly higher hit rates in the CYP inhibition assays, including CYP2C9 (banned cosmetic hit rate = 72%, *p* = 7.12 × 10^− 5^), CYP2C19 (69%, *p* = 1.46 × 10^− 2^), and CYP1A2 (69%, *p* = 4.02 × 10^− 2^), compared to the non-banned compounds (Fig. 5A). In addition, the banned cosmetics had significantly higher hit rates in the estrogen receptor agonist mode assay (tox21-er-luc-bg1-4e2-agonist-p4, banned cosmetic hit rate = 5%, *p* = 5.04 × 10^− 3^) and the androgen receptor antagonist mode assay (tox21-ar-bla-antagonist-p1, banned cosmetic hit rate = 15%, *p* = 1.12 × 10^− 2^) compared to the non-banned cosmetic ingredients (Fig. 5B). These pathways are essential in hormone regulation, which are known targets of endocrine disruptors such as phthalates and PFAS (classified as banned compounds). This analysis also revealed two new targets and pathways for the banned compounds, namely the constitutive androstane receptor (CAR, antagonist and agonist modes) and Keap1/Nrf2 (tox21-ks-are-p1, agonist mode), which were not reported previously to be affected by cosmetic ingredients. These data corroborate previous studies showing endocrine disruption by the banned compounds while also highlighting new pathways to study.

**Table 2:** Antagonist and agonist assays significantly altered by banned cosmetic compounds sorted by ascending *p*-value

Assay Name	Assay Mode	Target	Banned Cosmetic Hit Rate	*p*-value	PubChem Bioassay AID
tox21-p450-2c9-p1	Antagonist	CYP2C9	72.2%	7.12 × 10^− 5^	1671198
tox21-erb-bla-antagonist-p1	Antagonist	Estrogen receptor beta	18.9%	1.14 × 10^− 3^	1259396
tox21-ppard-bla-antagonist-p1	Antagonist	Peroxisome proliferator-activated receptor delta	5.75%	5.84 × 10^− 3^	743226
tox21-ar-bla-antagonist-p1	Antagonist	Androgen receptor	14.9%	1.12 × 10^− 2^	743063
tox21-p450-2c19-p1	Antagonist	CYP2C19	68.8%	1.46 × 10^− 2^	1671197
tox21-car-antagonist-p1	Antagonist	Constitutive androstane receptor	6.35%	2.68 × 10^− 2^	1224893
tox21-p450-1a2-p1	Antagonist	CYP1A2	68.8%	4.02 × 10^− 2^	1671199
tox21-er-luc-bg1-4e2-agonist-p4	Agonist	Estrogen receptor alpha	5.32%	5.04 × 10^− 3^	1259391
tox21-ks-are-p1	Agonist	Keap1/Nrf2	24.7%	7.51 × 10^− 3^	1919970
tox21-car-agonist-p1	Agonist	Constitutive androstane receptor	24.4%	1.73 × 10^− 2^	1224892
tox21-pgc-err-p1	Agonist	Estrogen-related receptor alpha	11.9%	3.23 × 10^− 2^	1259402
tox21-elg1-luc-agonist-p1	Agonist	ATAD5	8.91%	3.44 × 10^− 2^	720516
tox21-h2ax-cho-p2	Agonist	H2AX	12.9%	5.76 × 10^− 2^	1224896

**Fig. 6:**
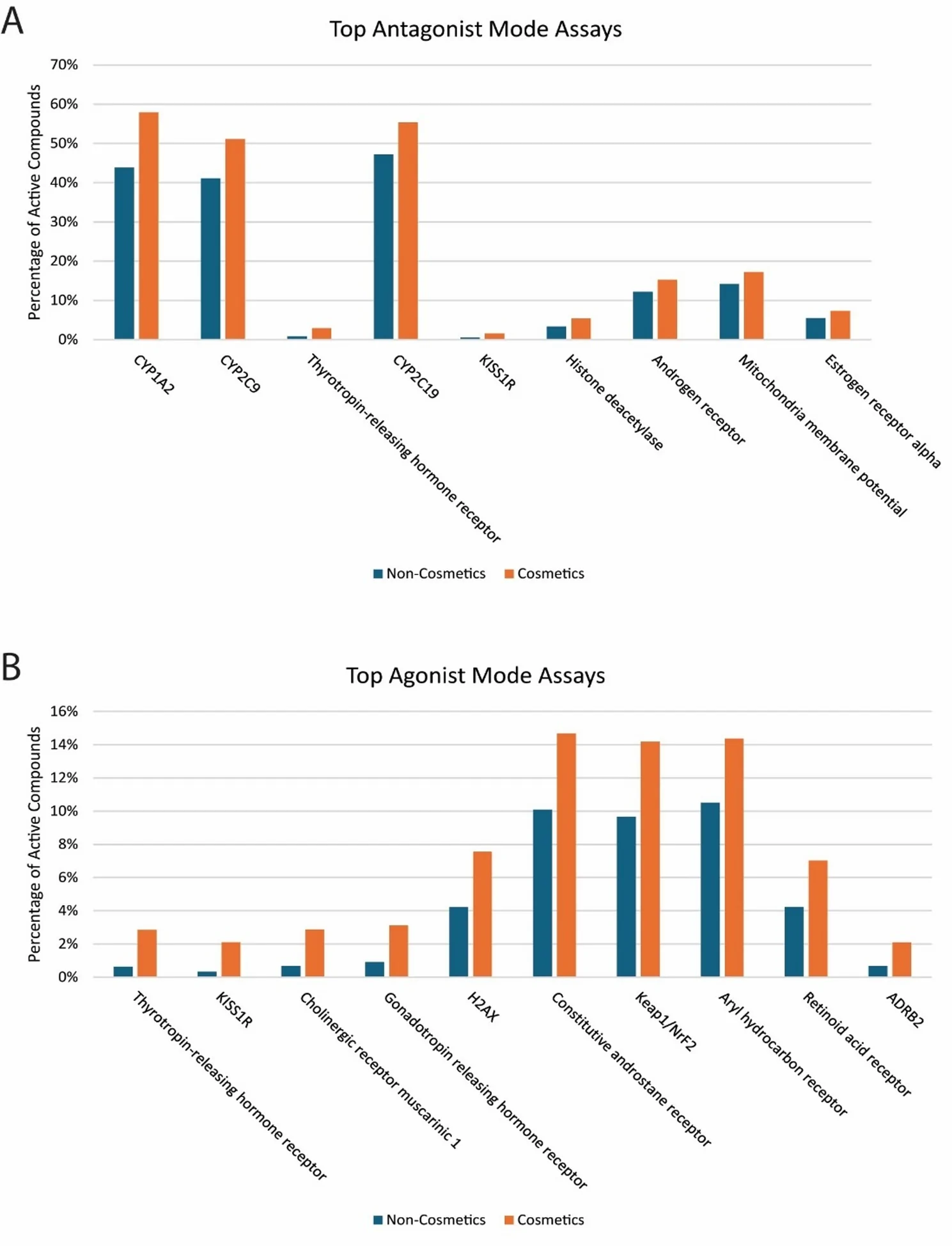
The percentage of active compounds in the cosmetic and non-cosmetic compound groups in the top antagonist mode (**A**) and agonist mode (**B**) assays significantly altered by cosmetic compounds

Next, the activity of all cosmetic labeled compounds (1,040) were compared to the non-cosmetic compounds (8,185) in the Tox21 10K library to determine the effect of cosmetic ingredients on different pathways. This comparison was done using a Fishers exact test comparing the number of active and inactive compounds in each category per assay. This analysis revealed 11 antagonist mode assays and 23 agonist mode assays that were significantly enriched with active compounds in the cosmetic ingredients. Multiple novel pathways were highlighted against which the cosmetic compounds were significantly more active compared to the non-cosmetic compounds. In terms of the antagonist mode assays, kisspeptin receptor (KISS1R) (2%, *p* = 2.04 × 10^− 3^) pathway was significantly affected by the cosmetic compounds. The agonist mode assays include the KISS1R (2%, *p* = 1.89 × 10^− 8^) as well as the CAR (15%, *p* = 1.34 × 10^− 4^), CHRM1 (3%, *p* = 3.53 × 10^− 8^), GnRH receptor (GnRHR) (3%, *p* = 4.37 × 10^− 7^), and Keap1/Nrf2 (14%, *p* = 1.55 × 10^− 4^). The top antagonist and agonist mode assays significantly affected by the cosmetic compounds are listed in Table 3 and shown in Fig. 6. These data show that, when considering the safety profile of cosmetic ingredients, these specific pathways may warrant further evaluation.

**Table 3:** List of top antagonist mode and agonist mode assays significantly altered by cosmetic compounds sorted by ascending *p*-value

Assay Name	Assay Mode	Target	Cosmetic Hit Rate	*p*-value	PubChem Bioassay AID
tox21-p450-1a2-p1	Antagonist	CYP1A2	57.9%	7.84 × 10^− 12^	1671199
tox21-p450-2c9-p1	Antagonist	CYP2C9	51.1%	3.44 × 10^− 7^	1671198
tox21-trhr-hek293-p1	Antagonist	Thyrotropin-releasing hormone receptor	2.90%	4.92 × 10^− 7^	1347038
tox21-p450-2c19-p1	Antagonist	CYP2C19	55.3%	6.75 × 10^− 5^	1671197
tox21-kiss1r-hek293-p2	Antagonist	KISS1R	1.58%	2.04 × 10^− 3^	1920068
tox21-hdac-p1	Antagonist	Histone deacetylase	5.37%	3.08 × 10^− 3^	1259388
tox21-ar-mda-kb2-luc-antagonist-p2	Antagonist	Androgen receptor	15.2%	2.07 × 10^− 2^	1259247
tox21-mitotox-p1	Antagonist	Mitochondria membrane potential	17.3%	2.53 × 10^− 2^	720637
tox21-er-luc-bg1-4e2-antagonist-p2	Antagonist	Estrogen receptor alpha	7.29%	4.78 × 10^− 2^	1259248
tox21-trhr-hek293-p1	Agonist	Thyrotropin-releasing hormone receptor	2.85%	1.76 × 10^− 8^	1347030
tox21-kiss1r-hek293-p2	Agonist	KISS1R	2.10%	1.89 × 10^− 8^	1920068
tox21-chrm1-p1	Agonist	Cholinergic receptor muscarinic 1	2.87%	3.53 × 10^− 8^	2061102
tox21-gnrhr-hek293-p2	Agonist	Gonadotropin releasing hormone receptor	3.11%	4.37 × 10^− 7^	1920067
tox21-h2ax-cho-p2	Agonist	H2AX	7.55%	4.10 × 10^− 5^	1224896
tox21-car-agonist-p1	Agonist	Constitutive androstane receptor	14.7%	1.34 × 10^− 4^	1224892
tox21-ks-are-p1	Agonist	Keap1/NrF2	14.2%	1.55 × 10^− 4^	1919970
tox21-ahr-p1	Agonist	Aryl hydrocarbon receptor	14.4%	1.03 × 10^− 3^	743122
tox21-rar-agonist-p1	Agonist	Retinoid acid receptor	7.02%	1.17 × 10^− 3^	1159553
tox21-adrb2-agonist-p1	Agonist	ADRB2	2.09%	1.32 × 10^− 3^	2061107

We further examined compound potencies in these assays, measured by their AC_50_s. Here we highlight two banned compounds, DEHP and perfluorooctane sulfonate (PFOS), and one non-banned compound, benzalkonium chloride, in some specific assays that they hit. DEHP was found to show activity in the following assays with various potencies: CYP2C9 (17.1 µM), CYP2C19 (30.3 µM), CYP1A2 (34.4 µM), and constitutive androstane receptor (CAR antagonist: 16.1 µM). PFOS was found active in the following assays: CYP2C9 (0.495 µM), CYP2C19 (33.6 µM), and Keap1/Nrf2 (64.8 µM). The non-banned compound, benzalkonium chloride, was shown to be active in multiple assays as well: Sonic Hedgehog pathway (0.55 µM) and the mitochondria toxicity (mitochondria membrane potential) assay (21 µM). While these AC_50_s are small and the data shows enzymatic activity, the compounds need to be studied further for their potential to cross physiological barriers, such as the blood brain barrier for neural targets like the Sonic Hedgehog pathway.

Since the Tox21 database has multiple sub-categories, the compounds labeled cosmetic ingredients were further compared to the categories of pesticide and drug compounds. When compared to pesticides, there were eight agonist and one antagonist assay that the cosmetic compounds were significantly more active. The antagonist assay was HDAC which is the Histone Deacetylase pathway while the eight agonist pathways contained five pathways related to estrogen and androgen disruption. In terms of drug compounds, the cosmetic compounds were significantly more active in eight antagonist and 14 agonist assays. The significant assays were mainly the assays highlighted in the cosmetic vs. non-cosmetic compounds. This makes sense since the drugs represent about 41% of the whole Tox21 10K library. Significant assays compared to each category are listed in Supplementary Table 2.

### Chemical structure analysis of banned and non-banned cosmetic ingredients

**Fig. 7:**
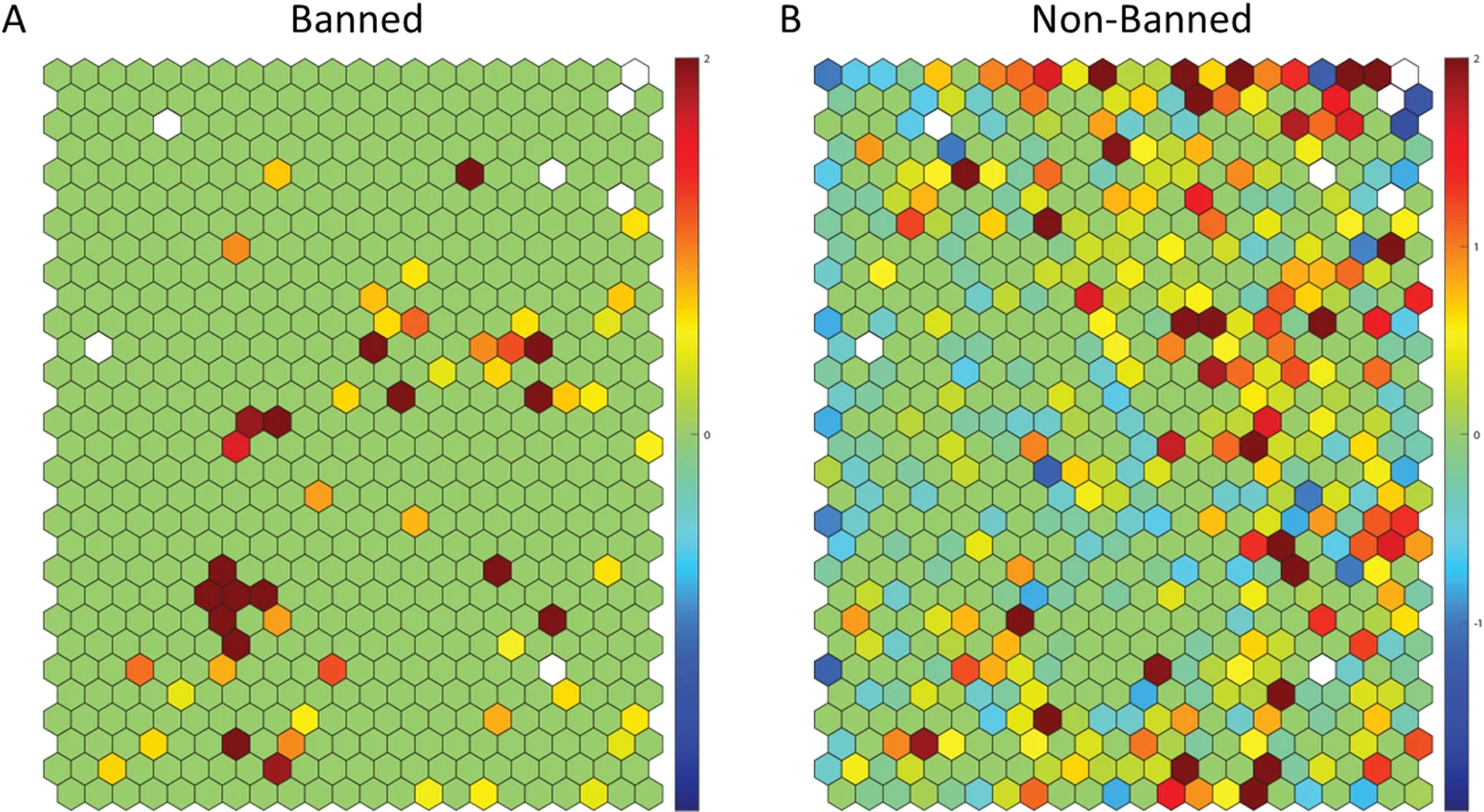
Self-organizing map (SOM) clustering of the Tox21 10K library based on chemical structure (ECFP4) similarity. In the heatmaps, each hexagon represents a cluster of structurally similar compounds. Hexagons are colored by the significance of enrichment (Fisher’s exact test p-value) of cosmetic compounds, banned (**A**) and non-banned (**B**). Red hexagons are enriched with cosmetic compounds whereas blue hexagons are deficient of cosmetic compounds compared to the library average. Darker shades of red or blue indicate higher levels of statistical significance (smaller *p*-value)

To evaluate the structural characteristics of the banned and non-banned cosmetic ingredients, the Tox21 10K compounds were clustered using the SOM algorithm based on structural similarity. Each cluster was evaluated for its enrichment of banned and non-banned cosmetic compounds (Fig. 7). Clusters enriched with cosmetic ingredients are colored in red, with darker shades of red indicating higher statistical significance. The Tox21 10K library was grouped into 653 clusters (660 clusters are visualized in the figure but 7 are empty white hexagons), 351 of which contain cosmetic compounds, with 49 clusters shared between the banned and non-banned compounds. The clusters are labeled as “column.row”, so k1.1 would be column 1 and row 1. A couple example clusters that are shared between the banned and non-banned cosmetics and are significantly enriched with cosmetic ingredients compared to the Tox21 10K library, are k23.19 and k29.9. Compounds in cluster k23.19 include aromatic amines that share chemical features such as bond: CN_amine_aromatic_generic, bond: CN_amine_ter-N_aliphatic, bond: CN_amine_ter-N_aromatic, bond: CN_amine_ter-N_aromatic_aliphatic, bond: CN_amine_ter-N_generic, bond: CN_amine_aliphatic_generic, and ring: aromatic_benzene. Both the banned compound N, N-dimethyl-p-phenylenediamine and non-banned compound Michler’s ketone fall into this cluster, which share the N, N-dimethylaniline feature. Cluster k29.9 contains single ring aromatic compounds, such as the banned compound toluene and non-banned compound aniline, and organo heavy metal compounds such as the banned compound phenylmercuric chloride and non-banned compound phenylmercury borate. While some of these are generic chemical features, cosmetic compounds that share these features with banned compounds should be investigated further to determine the potential toxicity of the non-banned compounds.

## Discussion

Exposure to cosmetics is unavoidable as they are designed not just for beauty purposes but are also utilized for hygiene and managing symptoms such as dry skin. Understanding how compounds used in cosmetics affect the body is a crucial part of assessing the levels of exposure acceptable and mitigating risks of exposure and accumulation of toxins over time. In this study, compounds used as cosmetic ingredients were analyzed to determine the pathways they alter and their potential toxicity compared to non-cosmetic ingredients. Multiple novel pathways of interest were highlighted in this study such as CAR (agonist), CHRM1 (agonist), KISS1R (agonist and antagonist), GnRHR (agonist), and Keap1/Nrf2 (agonist) that were shown to be significantly affected by the cosmetic ingredients compared to non-cosmetic compounds.

The skin is the largest organ and first line of defense against exposure to chemicals and the adverse effects associated with them. Given that the majority of cosmetics are meant to be “rubbed, poured, sprinkled, or sprayed on” the human body, it is important to understand how the chemicals interact with the body’s largest organ and primary barrier. The pathways significantly affected by cosmetic ingredients play pivotal roles in hormone regulation as well as immune functions. As previously noted, this analysis identified two targets, CAR and Keap1/Nrf2, as potentially disrupted by banned cosmetic compounds, with no prior reports of such associations in the literature. The constitutive androstane receptor (CAR) is regarded as a “master mediator” in xenobiotic disposition and detoxification but is also known to be crucial for homeostasis of glucose, lipids, and bile acids [46]. CAR has also been shown to be involved in cell proliferation, tissue regeneration/repair, and cancer development [46]. One compound that showed to be an active agonist of CAR is hydroquinone, which was historically used as a cosmetic but is now used as a skin bleaching agent available via prescription only.

Another pathway that was significantly affected by the cosmetic ingredients was the Keap1/Nrf2 (cosmetic hit rate = 14%, *p* = 1.55 × 10^− 4^) pathway which is an important part in the immune response to agitators on the skin. The Keap1/Nrf2 (tox21-ks-are-p1) assay has been shown to be a reliable test for skin sensitization [47]. Contact dermatitis is a common issue with prolonged use of cosmetics since it can lead to skin sensitization. For example, phenylmercuric acetate has been shown to cause skin allergies [48]. Phenylmercuric acetate is used as a preservative in shampoos and eye makeup even though it has been banned in paint and pesticides since 1990 due to its known toxicity [49]. The only restriction on its cosmetic use was the FDA’s limit on the amount of mercury acceptable in cosmetics until phenylmercuric acetate was banned at the state level (for multiple states) under the category of “mercury-containing”. Phenylmercuric acetate toxicity is related to its rapid metabolic breakdown into the mercuric ion and prolonged dermal exposure can lead to mercurialism [49]. Phenylmercuric acetate was shown to be one of the most cytotoxic cosmetic compounds with activity in 96.5% of the cell viability assays. Phenylmercuric acetate is not the only compound that is used in multiple products.

Another compound that is used in multiple ways is Zinc Pyrithione (ZPT). ZPT is used in antidandruff shampoos, carpet fibers, and preservatives. ZPT was highlighted in this study as an agonist of cholinergic receptor muscarinic 1 (CHRM1). CHRM1 gene is the gene that encodes muscarinic acetylcholine receptor M1 which is a receptor for acetylcholine. Acetylcholine plays a significant role in neuron communication and excitation at the neuromuscular junction, autonomic ganglia, and a neuromodulator in the brain [50]. Thus, if a compound is an agonist for CHRM1 it would result in an increased effect of acetylcholine on the body. Compounds like this, that target neuronal pathways, require more research in their potential to cross physiological barriers such as the blood brain barrier.

The potencies of the compounds (AC_50_) in relation to the relative levels of human exposure was compared for two prominent banned compounds, DEHP and PFOS, and one non-banned compound (benzalkonium chloride), to determine if the activity levels of these compounds are biologically relevant. Since DEHP is quickly metabolized, the amount of DEHP in the body is usually listed as the sum of its urinary metabolites. In a pregnant cohort, the amount of DEHP (determined by the sum of urinary metabolites) in the participants ranged from 0.032 to 0.41 ng/mL [51]. In terms of exposure risk, a pharmacokinetic model was built to predict external DEHP exposure based on plasma concentration and urinary excretion and found that the levels of exposure ranged from 0.52 to 157.52 µg/kg/day [52]. DEHP was found to show activities in the following assays with various potencies: CYP2C9 (17.1 µM), CYP2C19 (30.3 µM), CYP1A2 (34.4 µM), and constitutive androstane receptor (CAR antagonist: 16.1 µM). In terms of PFOS, one study on the association of perfluoroalkyl substances exposure with impaired lung function in children found that the range of the serum levels was 12.39–91.69 ng/mL [53]. In adults, one study found that the median PFOS ranged from 1.7 to 27.4 ng/mL (median of medians 7.7 ng/mL) across multiple studies [54]. The potencies of PFOS in the assays in which PFOS was found active were as follows: CYP2C9 (0.495 µM), CYP2C19 (33.6 µM), and Keap1/Nrf2 (64.8 µM).

A non-banned compound, benzalkonium chloride, was shown to be active in multiple assays including Sonic Hedgehog pathway (0.55 µM) and the mitochondria toxicity assay (21 µM). The Scientific Committee on Cosmetology calculated the margin of safety (MOS), which is the no observed adverse effect level divided by the systemic exposure dose, for benzalkonium chloride as 372, based upon daily bioavailability when used as preservatives, on skin (non-rinse), and hair products where the MOS must be greater than 100 to ensure human safety [55]. While the Cosmetic Ingredient Review (CIR) panel concluded in 2008 that it can be safely used at concentrations of up to 0.1% by weight [55], that means that consumers could be using multiple products that are formulated to that maximum level. Specifically, it can be used in products such as sunscreen and even though skin absorption may be limited, there are other routes of exposure such as oral and inhalation. When benzalkonium calcium was orally administered to rats, the lethal dose was reported to be 234–525 mg/kg and human alveolar epithelial (A549) cells that were incubated with benzalkonium chloride (1–10 µg/mL) for 24 h showed significant cytotoxicity [56].

Overall, several cosmetic compounds were found to be active across multiple different targets and pathways. Table 1 lists the top 20 most promiscuous and cytotoxic cosmetic ingredients based on hit rate when screened against the Tox21 assay panel. These compounds are primarily non-banned cosmetics, with nine of them being primarily used as preservatives. N, N-Dimethyl-N-benzyl-N-octadecylammonium chloride, benzalkonium chloride, and benzyl-C8-18-alkyldimethylammonium chloride are a few examples of preservatives that showed high cytotoxicity (active in > 95% cell viability assays) and promiscuity (active in > 84% Tox21 activity assays). For example, benzalkonium chloride targeted multiple biological targets and pathways, which include inhibition of the CHRM1 pathway (tox21-chrm1-p1) and Sonic Hedgehog pathway (tox21-shh-3t3-gli3-antagonist-p1), as well as disruption of the mitochondria membrane potential (tox21-mitotox-p1). This shows that it is not only the “active ingredients” in the cosmetics that need to be tested and followed up on but the preservatives and “inactive ingredients” as well. If a compound is promiscuous and cytotoxic, it could be affecting multiple pathways that are not commonly studied in toxicity assays. While the FDA has restricted or, in some instances, banned certain compounds for safety reasons, many compounds with uncharacterized toxicities continue to be used in cosmetics today.

A limitation of this study is the reliance on predetermined classification of compounds as “cosmetics” as designated in the EPA’s CPCat. Some compounds that were historically used in cosmetics (e.g., lindane) were banned over 40 years ago but still classified as a cosmetic ingredient in this study. The banned compounds in this study refer to the compounds currently being banned or are being considered to be banned at the state level so some historical compounds that were banned previously may still be labeled as “cosmetics” by the EPA even though the compound is no longer utilized. This study is also limited by the utilization of in vitro assays which do not account for the absorption, distribution, metabolism, or excretion of the compounds. In vitro assays do not account for long term exposure either. This study observes compounds at the cellular level absent of physiological barriers and changes. Exposure data for some of these compounds is limited because exposure is dependent on consumer use. Some compounds may not reach the biological level of activation unless bioaccumulation occurs or multiple products are used at their maximum permitted concentration. Understanding how cosmetic ingredients alter human physiology is critical for predicting and ultimately preventing adverse outcomes. The adverse effects of these cosmetic ingredients can start as early as fetal development in utero and can have lifelong consequences on the children exposed. In this study, multiple novel pathways were identified as potential targets of cosmetic ingredients. These include pathways that play a role in immune function, such as Keap1/Nrf2, and hormone regulation, such as GnRHR and KISS1R. Moreover, many of the promiscuous and cytotoxic compounds were found to be preservatives that are not banned. In conclusion, many non-banned cosmetic ingredients showed similar potential for toxicity as banned cosmetic ingredients but affected different pathways that were not previously identified. More research on the non-banned cosmetics highlighted in this paper is necessary to determine the long-term effects of exposure.

## Conclusions

In conclusion, cosmetics are unavoidable occurrences in today’s world. In the present study, we compared the bioactivity profiles of cosmetic ingredients and non-cosmetic compounds (not used in cosmetics), as well as the banned and non-banned cosmetic ingredients, across the Tox21 assays. These findings reveal distinct molecular hazard pathways for cosmetic ingredients to support safer development of cosmetics and further our mechanistic understanding of cosmetic ingredient toxicity. While the banned compounds have known mechanisms of toxicity, they aren’t limited in cosmetics except for in the specific states they are banned or are being banned. Some non-banned cosmetics share structural features and toxicity profiles similar to the banned compounds, suggesting that more research should be carried out on the toxicity in relation to the utilization of these compounds. This study highlighted the need to study cosmetic ingredients further, just because a compound isn’t banned doesn’t mean that it is completely safe. The idea of advancing next-generation risk assessment (NGRA) of cosmetic ingredients by harmonizing and utilizing new approach methodologies (NAMs) data and determining the best practices to advance cosmetic ingredient safety assessments in regulatory practice has recently expanded. Many NGRA studies do not aim to predict specific hazards associated with exposure but focus on ensuring that relevant exposures will have no meaningful bioactivity [57]. The International Cooperation on Cosmetics Regulation (ICCR) has created a team of scientists from industry and regulatory authorities to help advance the acceptance and define the best practices of utilizing NAMs in cosmetic safety assessments that align with the ICCR principles of NGRA for cosmetic ingredients [57]. Our analysis shows that the Tox21 dataset has the potential to play a central role in developing predictive, non-animal safety assessment frameworks for cosmetics.

## Supplementary Information

Below is the link to the electronic supplementary material.

Supplementary Material 1

Supplementary Material 2

Supplementary Material 3

## Data Availability

Data used in this study is available on the Tox21 website: https://tripod.nih.gov/fpubdata.

## Abbreviations

CAR: Constitutive Androstane Receptor
FDA: U.S. Food and Drug Administration
PFAS: Per- and polyfluoroalkyl substances
EPA: U.S. Environmental Protection Agency
CPCat: Chemical and Product Categories Database
DBP: Dibutyl Phthalate
DEHP: Diethylhexyl Phthalate
ZPT: Zinc Pyrithione
CHRM1: Agonist of cholinergic receptor muscarinic 1
NCATS: National Center for Advancing Translational Sciences
Tox21: Toxicology in the 21st Century
